# The use of dietary supplements and their association with blood pressure in a large Midwestern cohort

**DOI:** 10.1186/1472-6882-13-339

**Published:** 2013-11-28

**Authors:** Catherine A McCarty, Richard L Berg, Carla M Rottscheit, Richard A Dart

**Affiliations:** 1 Essentia Institute of Rural Health, 6AV-2 502 East Second Street, Duluth, MN 55805, USA; 2 Marshfield Clinic Research Foundation, Marshfield, WI, USA

**Keywords:** Blood pressure, Dietary supplements

## Abstract

**Background:**

There have been numerous studies assessing the association of diet and blood pressure but little is known about the association between less commonly used nutritional supplements and blood pressured. The purpose of this study was to quantify the use of dietary supplements and their potential association with blood pressure in a large population-based cohort of adults in the Midwest.

**Methods:**

The Personalized Medicine Research Project cohort was the population source for the current study. The current study includes subjects with Dietary History Questionnaire (DHQ) data available as well as at least one clinical blood pressure measurement recorded in their electronic medical record. After excluding extreme outlying measurements, median systolic and diastolic blood pressure measurements were calculated for each individual and were compared for subjects who did and did not report taking one of a list of 37 different supplements listed on the DHQ more than once per week over the previous 12 months.

**Results:**

9,732 subjects had both blood pressure and DHQ data available. They ranged in age from 18 to 98 years (mean 56 years) and 3,625 (37%) were male. Nine of 37 supplements showed evidence for association with blood pressure: coenzyme Q10, fish oil, iron, bilberry, echinacea, evening primrose oil, garlic, goldenseal and milk thistle. With the exception of the mineral iron, mean systolic and diastolic blood pressures were higher for users of the specific supplements than non-users.

**Conclusions:**

These results should not be interpreted as causal, nor can the direction of the association be assumed to be correct because the temporality of the association is unknown. We hope the observed significant associations will foster future research to evaluate blood pressure effects of dietary supplements.

## Background

One in three US adults has high blood pressure, defined as systolic blood pressure ≥140 mm Hg or diastolic blood pressure ≥ 90 mmHg or taking an antihypertensive medication or having been told at least twice by a physician or other health professional that one has high blood pressure [1]. High blood pressure is the single most important modifiable risk factor for stroke [2]. Less than half of people on antihypertensive medication have their blood pressure adequately controlled [3]. Among many causes of resistant hypertension (failure to achieve blood pressure control in patients who are adhering to full doses of appropriate three-drug regimen) are some over-the-counter dietary supplements such as ephedra, ma huang and bitter orange [4], while other dietary supplements such as coenzyme Q10, fish oil and garlic have been promoted for the management of hypertension [5].

The purpose of this study was to quantify the use of dietary supplements and their potential association with blood pressure in a large population-based cohort of adults in the Midwest. Existing data were employed for hypothesis generation about potential relationships between dietary supplement use and blood pressure. Where data exist, this can be a very cost effective approach, the first step being hypothesis generation, and the second step being hypothesis testing with a prospective study design that would allow determination of temporality.

## Methods

The Personalized Medicine Research Project (PMRP) [6] cohort was the population source for the current study. The PMRP is a population-based biobank in central Wisconsin with more than 20,000 adult participants aged 18 years and older, the vast majority of whom have received medical care through the Marshfield Clinic system of care for many years. Eligibility for PMRP included living in one of 19 Zip codes surrounding Marshfield, Wisconsin, and being a Marshfield Clinic patient aged 18 years and older with at least one contact with the Marshfield Clinic in the previous three years. The PMRP overall and the current sub-study were reviewed and approved by the Marshfield Clinic IRB. All subjects gave written informed consent to participate in PMRP, including access to their Marshfield Clinic medical records to classify phenotype.

In 2009, approximately six years after more than 18,000 subjects had been enrolled into PMRP, the National Cancer Institute Dietary History Questionnaire (DHQ) [7] was mailed to all subjects and was subsequently included for all subjects prospectively enrolled into the biobank [8]. The DHQ is a 36-page, self-administered food frequency questionnaire that includes 144 questions, with reference to usual intake in the past 12 months. Two questions about dietary supplements include a list of 37 supplements with instructions to mark use of specific supplements used more than once per week over the past 12 months.

Completed DHQs were returned by 63% of the living cohort for whom current addresses were available and who consented upon initial enrollment to future contact. The current sub-study of PMRP included subjects with DHQ data available as well as at least one clinical blood pressure measurement recorded in their Marshfield Clinic electronic medical record. The specific dietary supplements listed on the DHQ that were analyzed for the current study were: B-6, B-complex, brewer’s yeast, cod liver oil, coenzyme Q10, fish oil, folic acid/folate, glucosamine, hydroxytryptophan, iron, niacin, selenium, zinc, aloe vera, astragalus, bilberry, cascara sagrada, cat’s claw, cayenne, cranberry, Dong Kuai, echinacea, evening primrose oil, feverfew, garlic, ginger, ginkgo biloba, ginseng, goldenseal, grapeseed extract, kava, milk thistle, saw palmetto, St. John’s Wort, and valerian.

Blood pressure measurements for the sub-study cohort were retrieved as available from the Marshfield Clinic electronic medical record. Measurements associated with hospital stays were excluded as were blood pressure measurements recorded on days with emergency room or urgent care visits. For women, blood pressure measurements were excluded for the seven months prior to childbirth and for one month after delivery. After excluding extreme outlying measurements for individuals based on their personal range of blood pressure measurements, median systolic and diastolic blood pressure measurements were calculated for each individual using all results available within +/− 1 year of the dietary questionnaire (median 6 measures per individual, range 1–207). Similarly, the body mass index of each individual was obtained as the median of measures available in the electronic record within +/− 1 year of the dietary questionnaire (median 6 measures per individual, range 1–109).

Means of the per-person median systolic and diastolic blood pressure measurements were compared for subjects who did and did not report taking one of a list of 37 different supplements listed on the DHQ more than once per week over the previous 12 months. General linear models were used to adjust the comparisons for age, gender, body mass index, and current smoking status (as reported on baseline questionnaires). These variables were included in the linear models because of known association with blood pressure and because the data were relatively complete for the cohort, thus minimizing bias that could result from missing data. Data were analyzed with SAS® statistical software (Cary, NC). A p-value < 0.05 was required in this initial screening for statistically significant associations, without adjustment for multiple comparisons.

## Results and discussion

**Figure 1:**
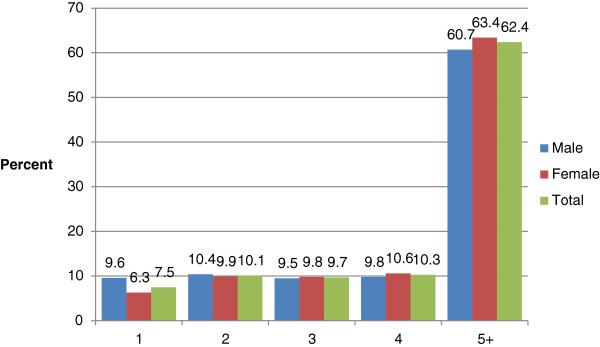
Gender-specific distribution of number of blood pressure measurements within one year of completion of the dietary history questionnaire.

**Figure 2:**
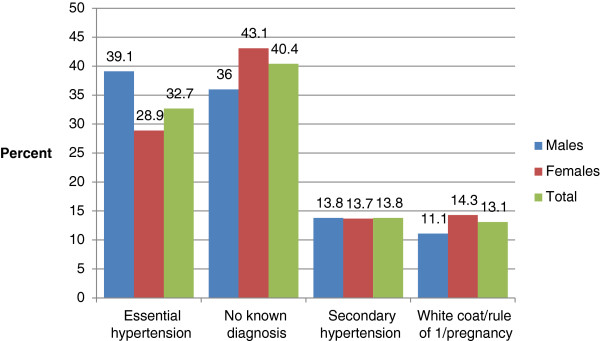
**Gender-specific distribution of hypertension diagnoses.** “White coat” is suspected elevated blood pressure associated with a clinical visit. “Rule of 1” refers to having only one diagnosis of hypertension in the medical record.

As of January 1, 2011, 19,981 subjects were enrolled in PMRP. Of these subjects 9,732 (49%) had both blood pressure and DHQ data available. They ranged in age from 18 to 98 years (mean 56 years) and 3,625 (37%) were male. The men were slightly older than the women (mean 59.5 for men and 54.2 for women), had slightly higher systolic and diastolic blood pressure measurements (Table 1). Body mass index and current smoking were similar among males and females. Although women had slightly more total blood pressure measurements in their medical records (Figure 1), men were more likely to have essential hypertension diagnosis (39.1% versus 28.9%, Figure 2). As expected given the definitions of hypertension, ever use of antihypertensive medications was higher in the essential hypertension and secondary cause of hypertension groups (Table 2).

**Table 1:** Gender-specific age, blood pressure, smoking and BMI at the time of biobank enrollment

**Measurement**	**Females, n = 6107**	**Males, n = 3625**	**Combined, n = 9732**
Age (mean, median, lower quartile, upper quartile, minimum, maximum)	54.4, 54.2, 42.0, 67.9, 18.4, 98.1	58.9, 59.5, 47.9, 71.8, 18.5, 96.3	56.1, 56.3, 44.1, 69.6, 18.4, 98.1
Systolic blood pressure (mean, median, lower quartile, upper quartile, minimum, maximum)	124.3, 123.5, 114.0, 133.0, 85.0, 201.0	127.2, 126.0, 119.0, 135.3, 80.0, 190.0	125.4, 124.0, 116.0, 134.0, 80.0, 201.0
Diastolic blood pressure (mean, median, lower quartile, upper quartile, minimum, maximum)	72.9, 72.0, 68.0, 78.5, 40.0, 107.0	73.4, 73.0, 68.0, 80.0, 42.0, 120.0	73.1, 72.5, 68.0, 79.0, 40.0, 120.0
Body mass index (mean, median, lower quartile, upper quartile, minimum, maximum)	29.6, 28.2, 24.2, 33.7, 14.6, 82.9	30.7, 29.7, 26.6, 33.6, 16.7, 79.9	30.0, 28.8, 25.1, 33.6, 14.6, 82.9
Number (%) current smokers	856 (14.0%)	524 (14.5%)	1380 (14.2%)

**Table 2:** Ever use of antihypertensive medication by hypertension diagnosis category

**Hypertension diagnosis category**	**Number ofsubjects indiagnosis category**	**Number (%) everused antihypertensivemedication**
No known hypertension	3934	556 (14.1%)
Essential hypertension diagnosis	3182	3080 (96.8%)
Secondary cause of hypertension	1340	1047 (78.1%)
“white coat”/rule of 1 (only a single diagnosis in the medical record)/pregnancy only	1275	387 (30.4%)

Self-reported use of dietary supplements varied by gender (Table 3). Women were more likely to report use of all supplements with the exception of brewer’s yeast, niacin, selenium, zinc, cayenne, ginkgo biloba, ginseng and saw palmetto.

**Table 3:** Weekly use of dietary supplements by gender in the PMRP

**Supplement**	**Males (n = 3625)**	**Females (n = 6107)**
	**No. (%) who reportedweekly use**	**No. (%) who reportedweekly use**
B6	71 (2.0)	229 (3.7)
B-complex	181 (5.0)	474 (7.8)
Brewer’s yeast	10 (0.3)	12 (0.2)
Cod liver oil	30 (0.8)	70 (1.1)
Coenzyme Q10	81 (2.2)	165 (2.7)
Fish oil	501 (13.8)	1072 (17.6)
Folic acid/folate	132 (3.6)	352 (5.8)
Glucosamine	382 (10.5)	745 (12.2)
Hydroxytryptophan	5 (0.1)	8 (0.1)
Iron	111 (3.1)	412 (6.7)
Niacin	96 (2.6)	103 (1.7)
Selenium	123 (3.4)	89 (1.5)
Zinc	149 (4.1)	217 (3.6)
Aloe vera	22 (0.6)	85 (1.4)
Astragalus	2 (0.1)	11 (0.2)
Bilberry	24 (0.7)	70 (1.1)
Cascara sagrada	0 (0.0)	7 (0.1)
Cat’s claw	3 (0.1)	3 (0.0)
Cayenne	22 (0.6)	29 (0.5)
Cranberry	48 (1.3)	194 (3.2)
Dong Kuai	1 (0.0)	14 (0.2)
Echinacea	31 (0.9)	141 (2.3)
Evening primrose oil	7 (0.2)	37 (0.6)
Feverfew	1 (0.0)	14 (0.2)
Garlic	147 (4.1)	254 (4.2)
Ginger	34 (0.9)	65 (1.1)
Ginkgo biloba	67 (1.8)	101 (1.7)
Ginseng	56 (1.5)	46 (0.8)
Goldenseal	6 (0.2)	33 (0.5)
Grapeseed extract	30 (0.8)	53 (0.9)
Kava	1 (0.0)	1 (0.0)
Milk thistle	19 (0.5)	36 (0.6)
Saw palmetto	107 (3.0)	6 (0.1)
Siberian ginseng	8 (0.2)	10 (0.2)
St. John’s wort	20 (0.6)	42 (0.7)
Valerian	4 (0.1)	29 (0.5)

A significant difference (after adjusting for age, gender, BMI and smoking) in systolic and/or diastolic blood pressure was observed between users and non-users of nine of the specific dietary supplements: coenzyme Q10, fish oil, iron, bilberry, echinacea, evening primrose oil, garlic, goldenseal and milk thistle (Table 4). The largest mean difference for systolic blood pressure was 5.3 mm Hg, a 4% difference, observed for evening primrose oil. This is a clinically meaningful difference. Smaller mean differences that were significantly different, such as the 0.5 mm Hg difference in mean diastolic blood pressure for fish oil, may not be clinically relevant and achieved statistical significance because of the large number of users of fish oil.

**Table 4:** Differences in mean systolic (SBP) and diastolic blood pressure (DBP) by supplement use for nine dietary supplements where significant blood pressure differences were observed (adjusted for age, gender, BMI ever use of antihypertensive medications and smoking)

**Supplement**	**Mean SBP**	**Mean SBP**	**SBP**	**Mean DBP**	**Mean DBP**	**DBP**
	**Non-users**	**Users**	**p-value**	**Non-users**	**Users**	**p-value**
Coenzyme Q10	125.3	127.3	0.011	73.1	73.6	0.314
Fish Oil	125.3	125.8	0.182	73.0	73.5	0.018
Iron	125.4	124.6	0.113	73.1	72.2	0.004
Bilberry	125.3	129.8	< 0.001	73.1	74.5	0.062
Echinacea	125.4	126.6	0.178	73.1	74.3	0.019
Evening primrose oil	125.4	130.1	0.008	73.1	74.3	0.355
Garlic	125.3	127.3	< 0.001	73.1	73.9	0.030
Goldenseal	125.4	130.2	0.012	73.1	75.1	0.110
Milk thistle	125.4	128.9	0.029	73.1	75.4	0.025

In the subset of subjects with no hypertension-related diagnoses, mean diastolic blood pressure was significantly lower for users of vitamin B6, cod liver oil, zinc and ginger and significantly higher for users of bilberry and goldenseal (Table 5). Mean systolic blood pressure was also significantly higher for users of bilberry and goldenseal.

**Table 5:** Differences in mean systolic (SBP) and diastolic blood pressure (DBP) by supplement use for six dietary supplements in subjects with no hypertension-related diagnoses where significant blood pressure differences were observed (adjusted for age, gender, BMI ever use of antihypertensive medications and smoking)

**Supplement**	**Mean SBP**	**Mean SBP**	**SBP**	**Mean DBP**	**Mean DBP**	**DBP**
	**Non-users**	**Users**	**p-value**	**Non-users**	**Users**	**p-value**
Vitamin B-6	118.6	118.6	0.980	72.0	70.6	0.043
Cod liver oil	118.6	116.1	0.163	71.9	69.3	0.034
Zinc	118.6	117.8	0.366	72.0	70.6	0.040
Bilberry	118.6	126.3	<.001	71.9	74.7	0.062
Ginger	118.6	117.6	0.505	71.9	69.5	0.017
Goldenseal	118.6	126.9	0.002	71.9	76.0	0.022

To our knowledge, this is one of the largest published studies of population-based estimates of dietary supplement use and their relationship to usual blood pressure. With the exception of the mineral iron, mean systolic and diastolic blood pressures were higher for users of the specific supplements which showed significant associations. These results should not be interpreted as causal, nor can the direction of the association be assumed to be correct because the temporality of the association is unknown. Rather, the significant associations should be considered hypothesis generating and used to foster future research with prospective study designs.

Coenzyme Q10, also known as ubiquinone, is an antioxidant which may act to decrease blood pressure by acting directly on vascular endothelium. Authors of a meta-analysis of clinical trials concluded that coenzyme Q10 has the potential to substantially lower blood pressure in people with hypertension [9], while the conclusion of a Cochrane review was that although the results of three clinical trials are clinically meaningful, the quality of the evidence did not justify use of Coenzyme Q10 for long-term management of hypertension [6].

Although not statistically significant to a very small sample size, one prior study in healthy volunteers found higher systolic blood pressure and lower diastolic blood pressure in subjects supplemented with echinacea in comparison with placebo [10].

Mean blood pressure was significantly lower in a study of normotensive rats that were injected with ginkgo biloba extract [11]. In a retrospective chart review of patients with normal tension glaucoma, 103 patients who took ginkgo biloba had significantly lower diastolic blood pressure in comparison with 97 patients who were not treated with gingko biloba [12]. In this same study, bilberry anthocyanins were associated with significantly higher systolic and diastolic blood pressures [12]. Suggested mechanisms of action relevant to blood pressure for ginkgo biloba include effects on blood circulation and for bilberry anthocyanin, antioxidant properties.

Similar to most previous studies [13,14], we found no association between use of ginseng and blood pressure.

The authors of two reviews of the influence of garlic on blood pressure [15] and cardiovascular morbidity and mortality in hypertensive patients [16] concluded that there was insufficient high quality evidence to support the use of garlic as an antihypertensive agent. It has been proposed that garlic may lower blood pressure through allicin, one of the bioactive compounds in garlic, by acting as a vasodilating agent.

There is a large body of evidence supporting an inverse association between blood pressure, hypertension and dietary intake of omega-3 fatty acids that are commonly found in fish. The authors of two meta-analyses published in 1993 concluded that there was dose–response blood pressure lowering effect of fish oil on blood pressure, perhaps through a vasodilation effect [17,18]. Two large studies, one with 4508 American adults aged 18–30 [19], and one with 4680 adults aged 40–59 from 17 samples in China, Japan, the United Kingdom and the United States [20] concluded that omega-3 fatty acid intake is inversely associated with blood pressure cross-sectionally [20] and inversely associated with the incidence of hypertension [19]. A meta-analysis comparing Mediterranean diets, typically high in fish but not necessarily low in total fat, to low-fat diets found that a Mediterranean diet has a greater inverse effect on blood pressure and other cardiovascular risk factors than a low-fat diet [21].

Iron is a trace element and essential nutrient. Several large epidemiological studies have demonstrated an inverse association between iron intake and blood pressure [22-24].

A number of data limitations need to be considered when reviewing these results, remembering that the data were intended for hypothesis generation, not hypothesis testing. First, the DHQs were collected after the majority of subjects had been recruited into the biobank and 4% of the cohort had died. To the extent that blood pressure was associated with mortality, there is the potential for bias in the observed associations between blood pressure and dietary supplement use. Second, temporality was not considered. Potential inverse associations could have resulted from changed behavior after a hypertension diagnosis. Blood pressure and height and weight measurements taken from medical records have the potential for inaccuracy because the measurements were not taken in a standardized fashion. We have attempted to minimize potential bias from inaccurate measurements by imposing rules about which data points to use or remove for individuals.

## Conclusions

These results should not be interpreted as causal, nor can the direction of the association be assumed to be correct because the temporality of the association is unknown. Despite these limitations, these data are intriguing and suggest areas for further research, where sufficient evidence does not already exist, into potential dietary supplements that could be used to lower blood pressure or for which use should be cautioned in people with hypertension.

## Competing interests

The authors declare that they have no competing interests.

## Authors’ contributions

CAM participated in the design of the study and drafted the paper. RLB conducted the statistical analyses. CR assisted in data collection and interpretation. RAD participated in the design of the study and the acquisition of data. All authors read and approved the final manuscript.

## Pre-publication history

The pre-publication history for this paper can be accessed here:

http://www.biomedcentral.com/1472-6882/13/339/prepub
